# A Sulfhydryl-Reactive Ruthenium (II) Complex and Its Conjugation to Protein G as a Universal Reagent for Fluorescent Immunoassays

**DOI:** 10.1371/journal.pone.0036086

**Published:** 2012-04-26

**Authors:** Jing-Tang Lin, Po-Chung Chen, Thirumani Venkatshwar Goud, Bor-Rong Huang, Tzu-Chau Lin, Jean-François Biellmann, Chien-Sheng Chen

**Affiliations:** 1 Graduate Institute of Systems Biology and Bioinformatics, National Central University, Jhongli City, Taoyuan Country, Taiwan; 2 Institute of Chemistry, Academia Sinica, Nankang, Taipei, Taiwan; 3 Department of Chemistry, National Central University, Jhongli City, Taoyuan Country, Taiwan; University of Birmingham, United Kingdom

## Abstract

To develop a fluorescent ruthenium complex for biosensing, we synthesized a novel sulfhydryl-reactive compound, 4-bromophenanthroline bis-2,2′-dipyridine Ruthenium bis (hexafluorophosphate). The synthesized Ru(II) complex was crosslinked with thiol-modified protein G to form a universal reagent for fluorescent immunoassays. The resulting Ru(II)-protein G conjugates were identified by sodium dodecyl sulfate-polyacrylamide gel electrophoresis (SDS-PAGE). The emission peak wavelength of the Ru(II)-protein G conjugate was 602 nm at the excitation of 452 nm which is similar to the spectra of the Ru(II) complex, indicating that Ru(II)-protein G conjugates still remain the same fluorescence after conjugation. To test the usefulness of the conjugate for biosensing, immunoglobulin G (IgG) binding assay was conducted. The result showed that Ru(II)-protein G conjugates were capable of binding IgG and the more cross-linkers to modify protein G, the higher conjugation efficiency. To demonstrate the feasibility of Ru(II)-protein G conjugates for fluorescent immunoassays, the detection of recombinant histidine-tagged protein using the conjugates and anti-histidine antibody was developed. The results showed that the histidine-tagged protein was successfully detected with dose-response, indicating that Ru(II)-protein G conjugate is a useful universal fluorescent reagent for quantitative immunoassays.

## Introduction

Biomolecule detection plays an important role in the biological research. Biosensing which uses biorecognition elements for detection is a rapid and easy method for biomolecule detection. The bioconjugation between detectable reagent and biorecognition elements is commonly used because of its higher sensitivity compared with label-free detecting system [1]. These detectable reagents include not exhaustively: fluorescence [2]–[4], chemiluminescence [5], radioactive isotopes [6]–[8], enzymes [9], nanocrystals [10] and liposomes [11].

Ru(II) polypyridine complex is one of the promising chemiluminescent reagents for biosensing due to high chemical stability and reversible reduction/oxidation reactivity [12], [13]. The electrogenerated chemiluminescence (ECL) using the ruthenium complexes have been widely developed for biosensor construction [5], [14], [15]. In addition, some ruthenium complexes are also fluorescent [16]. Several studies employed the ruthenium fluorophores as chelate or covalent stain for fluorescent protein detection in gel [17]–[19]. However, the development of fluorescent biosensing using a fluorescent ruthenium bioconjugate has not been reported.

We described here the synthesis of a novel sulfhydryl-reactive fluorescent ruthenium complex: 4-bromophenanthroline bis-2,2′-dipyridine Ruthenium bis (hexafluorophosphate), and its conjugation to protein G as a universal reagent for fluorescent immunoassays. Protein G is a bacterial cell wall protein originally isolated from group G *Streptococci*. and has universal IgG Fc-fragment binding ability [20]–[22]. It has great affinity with wide range of IgG subclasses and variety of mammalian species. Therefore, Ru(II)-protein G conjugate can be a great universal reagent useable for any immunoassays. In this study, the protein G was modified with *N*-succinimidyl S-acetylthioacetate (SATA), a heterobifunctional cross-linker, which provides sulfhydryl group to react with Ru(II) complex. The detection of antibody and recombinant histidine-tagged protein using Ru(II)-protein G conjugates were demonstrated, respectively.

## Results and Discussion

### Feature of Ru(II) complex

**Figure 1:**
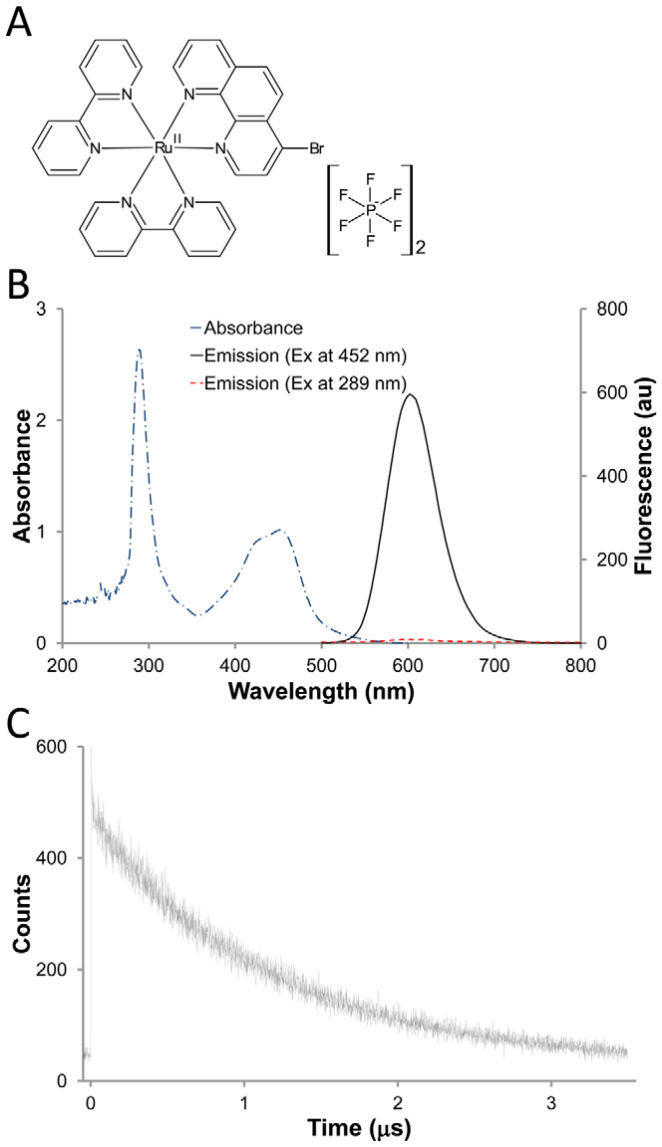
Features of Ru(II) complex. **A.** The chemical structure of Ru(II) complex, 4-bromophenanthroline bis-2,2′-dipyridine Ruthenium bis (hexafluorophosphate). **B.** The absorbance and emission spectra of Ru(II) complex. The absorbance spectrum (blue dot-dashed line) was scanned from OD_200_ to OD_600_. The major absorption peak was at 289 nm and minor peak was at 452 nm. Emission spectra were detected from 500 nm to 800 nm and excited at 452 nm (black solid line) and 289 nm (red dashed line), respectively. The Ru(II) complex using 289 nm excitation wavelength showed weak fluorescent signal. On the other hand, the Ru(II) complex using 452 nm excitation wavelength showed large fluorescent intensity and the emission peak wavelength of Ru(II) complex was at 602 nm. **C.** Fluorescence decay curve of Ru(II) complex.

The Ru(II) complex (Figure 1a) used in this study has two useful structural elements: the 4-bromophenanthroline group and the dipyridyl group. The 4-bromophenanthroline group is activated by the ligation to Ru undergoes an SnAr substitution reaction with the nucleophilic thiolate anion and thus provides the sulfhydryl-reactivity. The dipyridyl groups provide the spectral properties. The absorption peak was at 289 nm and 452 nm. Although 289 nm was the major absorption peak, the Ru(II) complex only show very weak fluorescence at the excitation of 289 nm. However, a strong fluorescent emission at 602 nm were observed at the excitation of 452 nm (Figure 1b). The Stokes shift of this Ru(II) complex is 150 nm, which is much larger than regular fluorescent dyes (10 to 30 nm). A large Stokes shift can increase the sensitivity of detection, decrease self-quenching and measurement error [23]. Also, interference filters can also be selected easily due to the large stoke shift. The fluorescent decay curve of Ru(II) complex was shown in Figure 1c. According to the curve, the lifetime of Ru(II) complex was calculated to be approximately 1.1 µs at room temperature, which is longer than regular fluorescence dyes. This feature allows the increase of biosensing sensitivity by avoiding the autofluorescence interference from some biomolecules. These results indicate that the Ru(II) complex synthesized in this study is an extraordinary fluorophore for biosensing.

### Conjugation of protein G with Ru(II) complex

**Figure 2:**
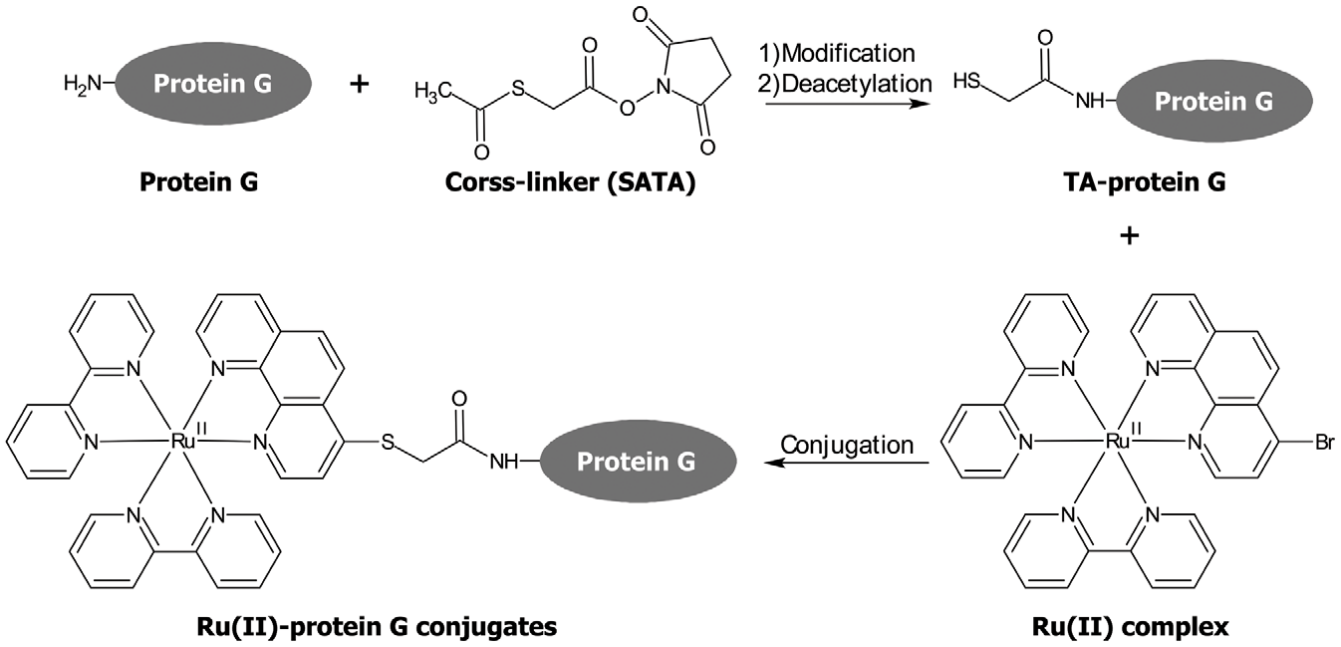
Schematics of conjugation between Ru(II) complex and protein G. The succinimide group of SATA reacted to primary amines of protein G and forms SATA modified protein G. Then, the SATA modified protein G was deacetylated by hydroxylamine. The resulting sulfhydryl modified protein G was conjugated with Ru(II) complex to form Ru(II)-protein G conjugates.

To conjugate protein with Ru(II) complex, the protein needs sulfhydryl group to react with the 4-bromophenanthroline group of Ru(II) complex. However, in nature, most sulfhydryl groups form disulfide bond for stabilizing tertiary protein structure and limited free sulfhydryl groups are available for Ru(II) complex conjugation. Although the internal disulfide bond can be broken by reducing reagent and sulfhydryl group of protein would be exposed, it will change the protein conformation and may lose the function. In this study, we used SATA as a cross-linker to provide additional reactive sulfhydryl group. As shown in Figure 2, the succinimide group of SATA reacts to primary amines of protein G. Then, the deacetylation of SATA modified protein G generated sulfhydryl group. Finally, the Ru(II) complex was conjugated onto sulfhydryl group of modified protein G.

### Identification of Ru(II)-protein G conjugates by SDS-PAGE

**Figure 3:**
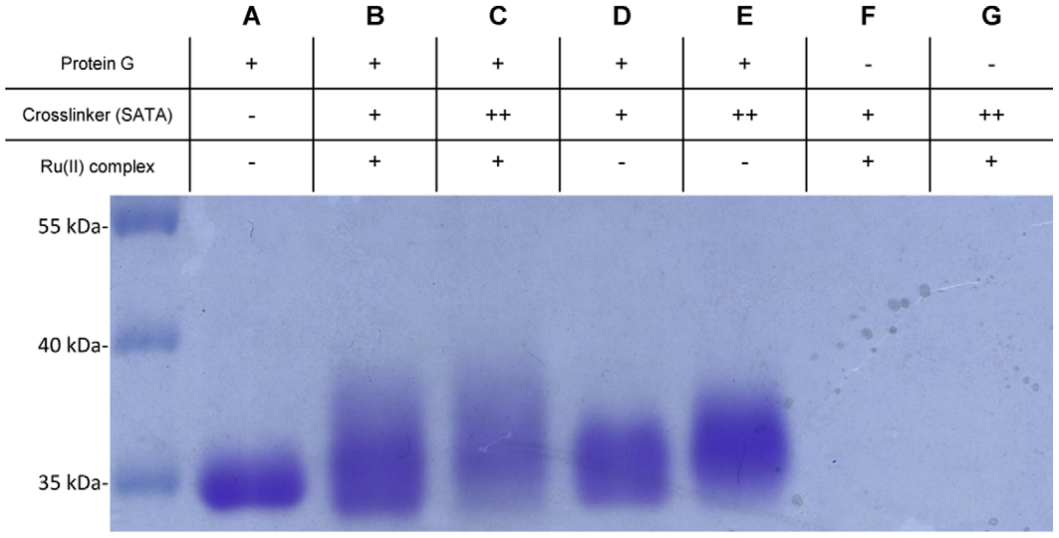
SDS-PAGE of protein G and Ru(II)-protein G conjugates. The Ru(II) complex was successfully conjugated to the SATA modified protein G and higher molar ratio of SATA to protein G provided higher conjugation efficiency. SATA (+) represents 25-fold molar ratio to protein G. SATA (++) represents 50-fold molar ratio to protein G. Negative control: conjugation without protein G (lane F and G). SATA-Ru(II) complex was expected to form in the negative control.

The SDS-PAGE was used to separate proteins according to the electrophoretic mobility for the estimation of molecular weight (Mw) of proteins and conjugations. As shown in Figure 3, the band of SATA modified protein G (lane D, E) showed larger band distribution toward higher Mw direction compared with the band of protein G (lane A). This result indicates that protein G had been successfully modified with the SATA. The band of 50-fold molar ratio of SATA to protein G (lane E) was even larger distributed toward higher Mw direction than the band of 25-fold molar ratio of SATA to protein G (lane D), which indicates that the higher molar ratio of SATA to protein G, the more SATA molecules were modified onto protein G. Similarly, the band of Ru(II)-protein G conjugates (lane B, C) showed broader band distribution toward higher Mw direction compared with the band of SATA modified protein G (lane D, E), indicating that Ru(II) complex was successfully conjugated to the thiol-modified protein G. The band of 50-fold molar ratio of SATA to protein G with Ru(II) complex (lane C) showed larger band distribution and the band shifted to higher molecular weight position compared with the band of 25-fold molar ratio of SATA to protein G with Ru(II) complex (lane B). This also suggested that higher molar ratio of SATA to protein G provides higher conjugation efficiency.

### Absorbance and emission spectra of Ru(II)-protein G conjugates

**Figure 4:**
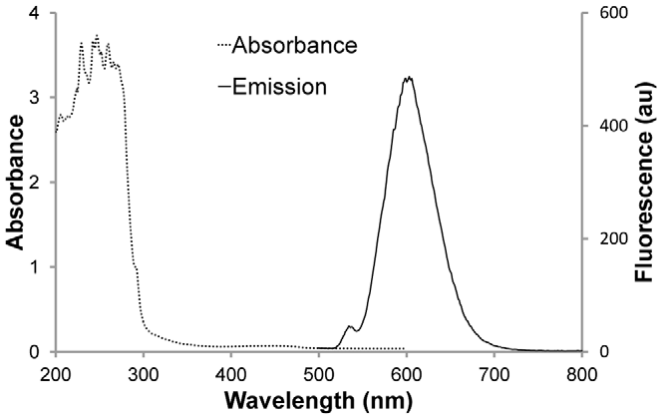
The absorbance and emission spectra of Ru(II)-protein G conjugates. The absorbance spectrum (dotted line) was scanned from OD_200_ to OD_600_. The emission spectrum (solid line) was scanned from 500 nm to 800 nm using 452 nm excitation wavelength.

To investigate conjugation effect of protein G with Ru(II) complex on photophysical property, the resulting Ru(II)-protein G conjugates was conducted with spectroscopy. As shown in Figure 4, the absorption spectrum of the Ru(II)-protein G conjugates showed a typical protein absorption peak around 280 nm, but the Ru (II) complex absorption at 452 nm was relative weak. Nevertheless, it still showed a typical fluorescence spectrum of the Ru (II) complex when the Ru(II)-protein G conjugates was excited at 452 nm. The fluorescent peak wavelength of Ru(II)-protein G conjugates was at 602 nm, which is similar to the Ru (II) complex. These results indicated that Ru(II)-protein G conjugates was still a feasible fluorophore after conjugation.

### IgG-binding assay of Ru(II)-protein G conjugates

**Figure 5:**
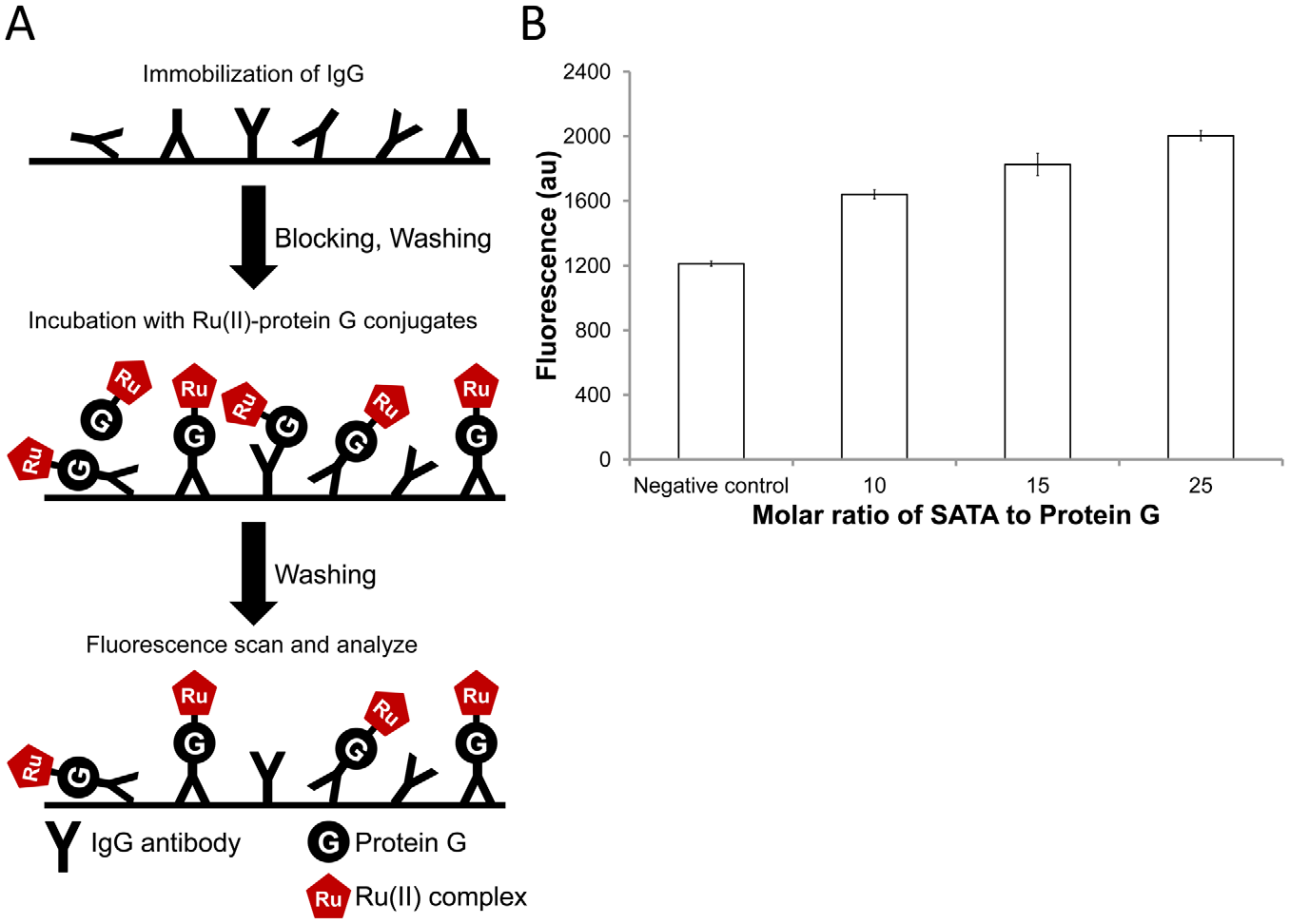
IgG binding assay of Ru(II)-protein G conjugates. **A.** Schematic of IgG-binding assays of Ru(II)-protein G conjugates. Normal sheep IgG was immobilized on the 96 well plate, and then Ru(II)-protein G conjugates bound to the Fc region of IgG. **B.** Effect of molar ratios of SATA to Protein G for conjugation were tested: 10, 15 and 25-fold. Negative control: Ru(II) complex without Protein G.

To examine the IgG binding ability of Ru(II)-protein G conjugates, IgG-binding assay was developed in this study. As shown in Figure 5a, polyclonal antibody IgG was first immobilized on 96 well plate. Then, Ru(II)-protein G conjugates were added for the detection of the immobilized IgG Fc fragment. As shown in Figure 5b, compared with negative control (Ru complex without Protein G), the Ru(II)-protein G conjugates showed higher fluorescent intensity at excitation wavelength of 485 nm and emission wavelength of 620 nm. It demonstrated that Ru(II)-protein G conjugates are capable of binding the Fc region of IgG and useful for immunoassays. The 10-, 15- and 25-fold molar ratio of SATA to protein G were tested. The fluorescent intensity increased with the increase of SATA to protein G in IgG binding assay. This result indicated that higher ratio of SATA to protein G provides higher conjugation efficiency, which is consistent with the SDS-PAGE result.

### Application of Ru(II)-protein G conjugates in fluorescent immunoassay

**Figure 6:**
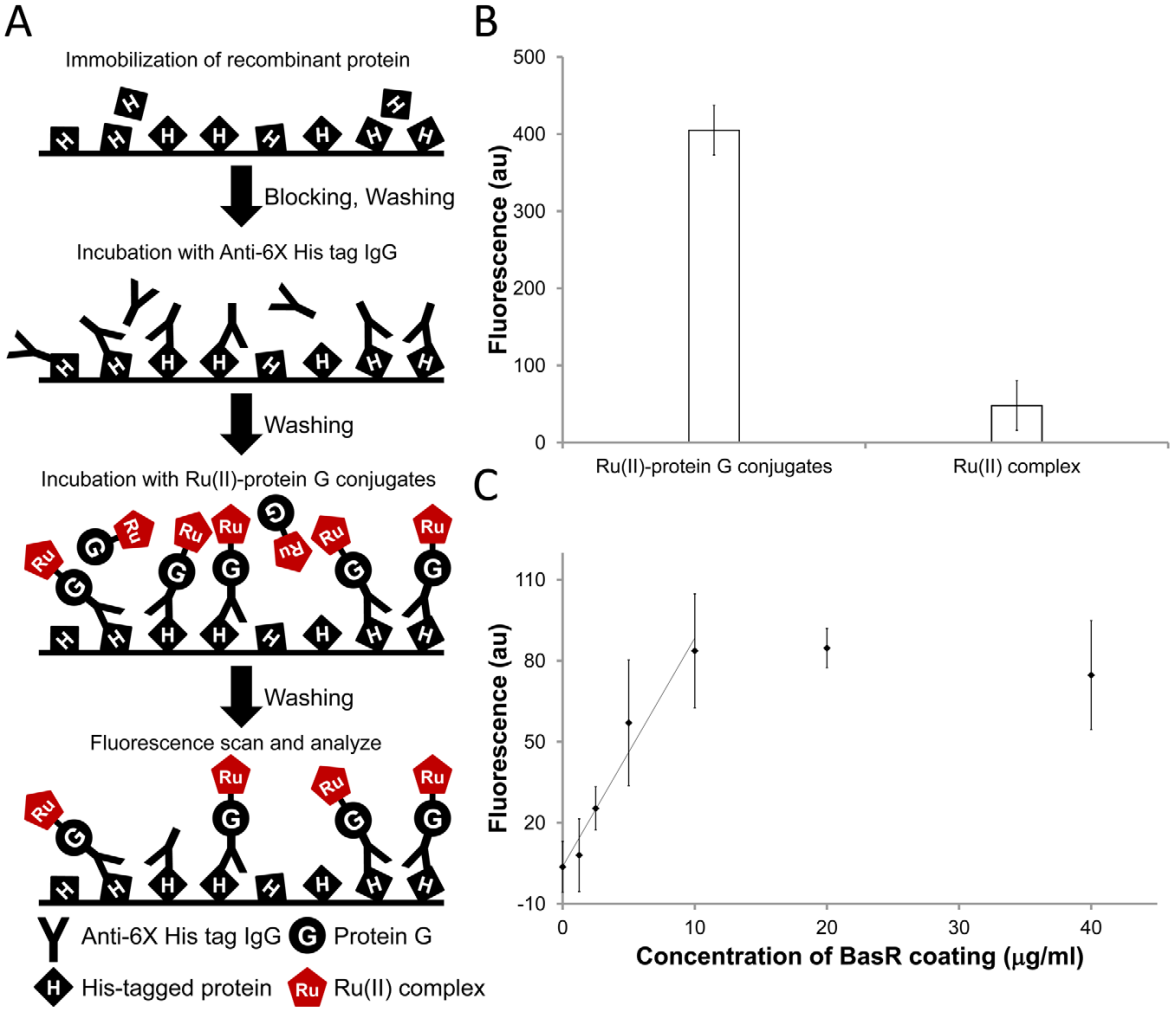
Detection of purified recombinant proteins by Ru(II)-protein G conjugates. **A.** Schematic of Ru(II)-protein G conjugates for detecting histidine-tagged protein. The purified histidine-tagged protein BasR was first immobilized on the 96 well plate and then recognized by anti-His antibody. Finally, the Ru(II)-protein G conjugates bound to the Fc region of anti-His antibody. **B.** Comparison of Ru(II)-protein G conjugates and Ru(II) complex for detecting histidine-tagged protein BasR. Ru(II)-protein G conjugates showed approximate 8-fold fluorescent signal compared with Ru(II) complexes (negative control) in the assay. **C.** Dose response of the histidine-tagged recombinant protein. The linear dynamic range of was from 0 to 10 µg/ml (R^2^ = 0.96).

To demonstrate the feasibility of Ru(II)-protein G conjugates for immunoassays, we employed the conjugates for the detection of the most common recombinant protein, histidine-tagged protein. As shown in Figure 6a, the purified histidine-tagged recombinant protein was first immobilized on 96 well plate. Then, anti-6X His tag antibody was added to bind the immobilized histidine-tagged recombinant protein BasR. The Ru(II)-protein G conjugates was then added to bind the antibody as a universal signal reporter. As shown in Figure 6b, the fluorescent intensity of Ru(II)-protein G conjugates was approximate 8-fold to Ru(II) complex (negative control) indicating that the Ru(II)-protein G conjugates is a good universal reagent for fluorescent immunoassays. Furthermore, seven concentrations (including 0, 1.25, 2.5, 5, 10, 20 and 40 µg/ml) of recombinant protein BasR were used to observe the dose response. As shown in Figure 6c, the linear dynamic range of BasR was from 0 to 10 µg/ml (R^2^ = 0.96) and dose response curve showed saturation after 10 µg/ml. These results demonstrated that Ru(II)-protein G conjugates were successfully applied for a quantitative immunoassay. It is often important to measure the concentration of recombinant protein for further biological research.

### Conclusion

We successfully synthesized a novel sulfhydryl-reactive Ru(II) complex, 4-bromophenanthroline bis-2,2′-dipyridine Ruthenium bis (hexafluorophosphate) with fluorescence. The Ru(II) complex were bioconjugated with protein G by the aid of SATA to become a universal fluorescent reagent for immunoassays. Finally, it was demonstrated that Ru(II)-protein G conjugates were successfully used for the quantitative detection of histidine-tagged protein.

## Materials and Methods

### Synthesis of 4-bromophenanthroline bis-2,2′-dipyridine Ruthenium bis (hexafluorophosphate)

The ruthenium complex [(2,2-bpy)_2_Ru(4-bromo-1,10-phenanthroline)]^2+^(PF_6_)^−^_2_ was prepared by reaction of bis(2,2-bipyridine)Ru(II) dichloride dihydrate [(bpy)_2_RuCl_2_, 2H_2_O] with 4-bromo-1,10-phenanthroline [24] as described for an analog compound [25]. The 4-bromoRu complexes crystallized from water/methanol/acetone (v 1/2/2) was obtained with a yield of 75–80%. UV (λmax): 450 nm (ε 19000), 286 (ε 87000), 267 (ε 84000). IR (CH3CN): 2921, 2851, 2354, 1595, 1258, 840, 728 cm−1. 1H NMR (CD3CN, 500 MHz): δ 8.53 (d, J = 8.0 Hz, 1H), 8.49 - 8.43 (m, 5H), 8.35 (d, J = 9.2 Hz, 1H), 8.12 - 8.09 (m, 3H), 8.03 – 8.0 (m, 3H), 7.88 (d, J = 5.6 Hz, 1H), 7.82 (d, J = 5.6 Hz, 2H), 7.78 – 7.74 (m, 1H), 7.57 (d, J = 5.6 Hz, 1H), 7.51 (d, J = 5.6 Hz, 1H), 7.44 - 7.43 (m, 2H), 7.23 – 7.20 (m, 2H). 13C NMR (CD3CN, 125 MHz): δ 157.86, 157.59, 157.47, 153.90, 152.83, 152.66, 152.61, 148.45, 148.10, 138.61, 138.48, 137.61, 131.80, 131.40, 130.31, 130.21, 128.23, 128.11, 128.03, 127.32, 127.25, 124.93, 124.85. Mass spectrum (FABMS): m/z 817.5 (M+H); HRMS Calcd. for C32H24BrF6N6PRu 817.5034, found 817.5028.

### Conjugation of Ru(II) complex and protein G

Nineteen µl of 3, 4.5, 7.5, or 15 mM SATA (Thermo) in DMSO (Sigma) was added into 300 µl of 19 µM protein G in 50 mM, pH 8.0 borate buffer (10-, 15-, 25-, or 50-fold of molar ratio of SATA to protein G) at ambient temperature, respectively. After 1 hour, the unreacted *N*-succinimide group of SATA was then quenched by adding 57 µl of Tris solution (250 mM Tris-base, pH 8.0) for 15 min. The sulfhydryl group was generated by 38 µl of deacetylation solution (500 mM hydroxylamine, 250 mM EDTA, pH 8.0) for 2 hours. One hundred and four µl of Ru(II) complex in DMSO was incubated with the deacetylated SATA modified protein G solution at ambient temperature for 3.5 hours. The unreacted deacetylated SATA modified protein G was quenched by 29 µl of 100 mM *N*-ethylmaleimide in 50 mM, pH 8.0, borate buffer for 15 min. Purification was carried out after conjugation by centrifugal ultrafiltration (Vivaspin 500, Sartorius Stedim Biotech).

### Absorbance and emission spectra measurement

The absorbance and emission spectra of the Ru(II) complex and purified Ru(II)-protein G conjugates prepared above were determined, respectively. The absorbance spectrum (200 nm to 600 nm) was taken with a Synergy™ 2 (BioTek®) spectrometer and the emission spectrum was taken with a HITACHI F-4500 fluorometer (excitation wavelength at 452 nm and emission wavelength from 500 nm to 800 nm, emission slit at 5 nm). The PMT voltage of fluorescent detection in Figure 1b and Figure 3 were set at 700 V and 950 V, respectively.

### Fluorescence decay curve

The fluorescence decay profile of Ru(II) complex was measured by FluoroMax®-4 Spectrofluorometer at ambient temperature (excitation wavelength at 404 nm and emission wavelength at 602 nm, emission slit at 1 nm). The lifetime of Ru(II) complex was calculated by decay analysis software, DAS6 (HORIBA Scientific).

### Sodium dodecyl sulfate-polyacrylamide gel electrophoresis (SDS-PAGE)

The protein G, SATA modified protein G, SATA-Ru(II) complex, and purified Ru(II)-protein G conjugates (25- and 50-fold molar ratio of SATA to protein G with Ru(II) complex) were first treated with reducing and denaturing reagents (0.25 M DTT, 10% SDS) at 95°C for 10 min, respectively. Then, they were conducted in 10% polyacrylamide gel with electrophoresis at 80 V. The gel was stained with coomassie blue solution (0.1% coomassie brilliant blue R-250, w/v) for 16 hours. After electrophoresis, the gel was treated with a destaining solution (50% methanol, v/v; 10% acetic acid, v/v) 5 times for 30 min. The gel image was scanned by ArtixScan 1800f (Microtek).

### IgG binding assay using Ru(II)-protein G conjugates

A solution (100 µl) of 20 µg/ml normal sheep IgG (abcam®) in 1× phosphate buffered saline buffer (1× PBS: 10 mM Na_2_HPO_4_, 1.76 mM KH_2_PO_4_, 0.14 M NaCl, 2.6 mM KCl, pH 7.4) was immobilized on the Nunc-Immuno™ plates wells for 2 hours. Then, blocking buffer (1% bovine serum albumin, 0.25 M Tris-base, 1.4 M NaCl, pH 7.4) exchanged normal sheep IgG solution for 2 hour blocking. After removing blocking buffer, Ru(II)-protein G conjugates (100 µl) were added into each well and incubated with immobilized normal sheep IgG for 1 hour. Each well was then washed three times for 10 min each in blocking buffer on an orbital shaker. Finally, the solution in each well was removed completely, and Synergy™ 2 (BioTek®) reader was used to read fluorescent intensity, setting excitation wavelength at 485 nm and emission wavelength at 620 nm with dichroic mirror at 510 nm. All the experiments were conducted at ambient temperature.

### Purification of recombinant histidine-tagged protein

The recombinant histidine-tagged protein BasR transcriptional regulator from *E. coli* K12 was purified by Ni-NTA column purification. Briefly, the BasR *E. coli* clones (from ASKA collection constructed by Dr. Mori and co-workers [26]) was incubated in 2× LB medium containing 30 µg/ml of chloramphenicol at 37°C for overnight. After overnight incubation, the culture was then diluted with 2× LB to OD_595_ value of 0.1. When the OD_595_ was 0.3, the recombinant histidine-tagged protein BasR was induced with 0.5 mM of isopropyl β-D-thiogalactoside at 30°C for 4 hours. Then, the culture was centrifuged at 4°C to obtain cell pellets. For protein purification, the cell pellets were mixed with lysis buffer (50 mM NaH_2_PO_4_, 300 mM NaCl, 30 mM imidazole, CelLyticB, 1 mg/ml lysozyme, 50 units/ml proteinase inhibitor cocktail and 1 mM PMSF) and Ni-NTA resin at 4°C for 2.5 hours incubation. The protein-resin complexes were washed five times with wash buffer I (50 mM NaH_2_PO_4_, 300 mM NaCl, 20% glycerol, 20 mM imidazole and 0.1% Tween 20, pH 8.0) and wash buffer II (50 mM NaH_2_PO_4_, 150 mM NaCl, 30% glycerol, 30 mM imidazole and 0.1% Tween 20, pH 8.0). Finally, recombinant histidine-tagged protein was eluted using elution buffer (50 mM NaH_2_PO_4_, 150 mM NaCl, 30% glycerol, 300 mM imidazole and 0.1% Tween 20, pH 7.5). The quantitation of recombinant protein was carried out by BCA™ protein assay kit (Thermo) and molecular weight was confirmed by SDS-PAGE after recombinant protein purification.

### Detection of recombinant histidine-tagged protein using Ru(II)-protein G conjugates

A solution (100 µl) of 40 µg/ml recombinant histidine-tagged BasR protein in 1× PBS buffer was first immobilized on Nunc-Immuno™ Plates for 2 hours. Then, blocking buffer replaced BasR protein solution and incubated for 1 hour. After removing blocking buffer, a solution (100 µl) of 15 µg/ml anti-6X His tag monoclonal antibody (abcam®) in 1× PBS buffer, pH 7.4, was added into each well for 1 hour incubation on an orbital shaker. Each well was then washed and rinsed three times for 10 min in blocking buffer. The Ru(II)-protein G conjugates solution (100 µl) was added to interact with Fc region of anti-6X His tag monoclonal antibody for 1 hour incubation. The wells were then washed using three times for 10 min in blocking buffer. Synergy™ 2 reader was also used to measure fluorescence intensity. All the experiments were conducted at ambient temperature.
